# Cells release subpopulations of exosomes with distinct molecular and biological properties

**DOI:** 10.1038/srep22519

**Published:** 2016-03-02

**Authors:** Eduard Willms, Henrik J. Johansson, Imre Mäger, Yi Lee, K. Emelie M. Blomberg, Mariam Sadik, Amr Alaarg, C.I. Edvard Smith, Janne Lehtiö, Samir EL Andaloussi, Matthew J.A. Wood, Pieter Vader

**Affiliations:** 1 Department of Physiology, Anatomy and Genetics, University of Oxford, Oxford, OX1 3QX, United Kingdom; 2 Cancer Proteomics Mass Spectrometry, Science for Life Laboratory, Department of Oncology-Pathology, Karolinska Institutet, Stockholm, SE-171 21, Sweden; 3 Institute of Technology, University of Tartu, Tartu, 50411, Estonia; 4 Department of Laboratory Medicine, Karolinska Institutet, Stockholm, SE-141 57, Sweden; 5 Department of Clinical Chemistry and Haematology, University Medical Center Utrecht, Utrecht, 3584 CX, The Netherlands

## Abstract

Cells release nano-sized membrane vesicles that are involved in intercellular communication by transferring biological information between cells. It is generally accepted that cells release at least three types of extracellular vesicles (EVs): apoptotic bodies, microvesicles and exosomes. While a wide range of putative biological functions have been attributed to exosomes, they are assumed to represent a homogenous population of EVs. We hypothesized the existence of subpopulations of exosomes with defined molecular compositions and biological properties. Density gradient centrifugation of isolated exosomes revealed the presence of two distinct subpopulations, differing in biophysical properties and their proteomic and RNA repertoires. Interestingly, the subpopulations mediated differential effects on the gene expression programmes in recipient cells. In conclusion, we demonstrate that cells release distinct exosome subpopulations with unique compositions that elicit differential effects on recipient cells. Further dissection of exosome heterogeneity will advance our understanding of exosomal biology in health and disease and accelerate the development of exosome-based diagnostics and therapeutics.

Extracellular vesicles (EVs) are heterogeneous populations of endogenous nano-sized cell-derived membrane vesicles released by eukaryotic and prokaryotic cells^1^,^2^. EVs play an important role in intercellular communication via transfer of their biological content, which consists of proteins, lipids and nucleic acids, between cells^3^,^4^,^5^,^6^. As a growing body of evidence suggests that EVs are involved in physiological as well as pathological processes, interest in the biological roles of EVs and their clinical application is growing^7^.

EVs are lipid bilayer enclosed membrane vesicles ranging from 30 nm to 2,000 nm in diameter. Although there is ongoing discussion in the field, it seems that EVs can be categorized into three main classes, based on their biogenesis pathways: exosomes, microvesicles and apoptotic bodies^8^. Microvesicles (MVs) originate from the cell surface, where they are released by direct outward budding of the plasma membrane. Their heterogeneous size ranges from 50 nm to 1,000 nm in diameter. Apoptotic bodies are released through outward blebbing and fragmentation of the cell membrane of apoptotic cells, and have a broad size range of 50–2,000 nm in diameter.

Exosomes are derived from the endolysosomal pathway and are formed within multivesicular bodies (MVBs). They are released by cells upon fusion of MVBs with the plasma membrane^9^. In contrast to MVs, exosomes are presumed to represent a more homogenous population of EVs, ranging in size from 30 nm to 120 nm in diameter. Sorting of cargo into exosomes involves specific proteins associated with the endosomal sorting complex required for transport (ESCRT), such as ALG-2-interacting protein X (ALIX) and tumour susceptibility gene 101 protein (TSG101)^10^. As a result, ALIX and TSG101 are commonly used as marker proteins for exosomes.

Despite their seemingly homogenous characteristics, exosomes mediate a wide spectrum of effects on recipient cells^7^,^11^,^12^. This could indicate that exosomes are highly multifunctional vesicles, or that cells release exosomes characterized by heterogeneity, i.e. subpopulations of exosomes display distinct compositions and/or functions. Studies on MVB sorting mechanisms, content and composition support the potential heterogeneous nature of exosomes^9^,^13^. For example, exosome formation inside MVBs can rely on ESCRT-dependent and independent pathways^14^,^15^. Therefore, alterations in these pathways could affect MVB dynamics and the subsequent release of subpopulations of exosomes. Analysis of the stoichiometry of miRNAs and exosomes suggests that most individual exosomes do not carry biologically significant numbers of miRNA copies^16^. Given the increasing number of reports showing functional transfer of miRNAs via exosomes^17^,^18^,^19^, and taking into account that exosome transfer can be a highly selective and infrequent event, these findings may point towards the presence of miRNA-rich subpopulations of exosomes. Work performed by Palma *et al*. showed differential packaging of miRNAs and subsequent release of distinct subpopulations of exosome-like vesicles by cancerous cells as compared to normal cells. Increased release of one of the subpopulations possibly resulted from changes in sub-endocytic pathways^20^. Others have shown that cellular activation can alter the dynamics of exosome release by increasing the release of specific populations of vesicles^21^.

These observations support the idea that exosomes represent a heterogeneous population of vesicles. Identification and separation of exosome subpopulations is of great importance for studies on exosome biology and function. Here, we were able to separate two major distinct subpopulations of exosomes from different cell sources. Exosome subpopulations isolated from melanoma cells were subsequently characterized for their biophysical characteristics and molecular composition. We further evaluated whether exosome subpopulations have different biological effects on recipient cells. The results from this study may have important implications for future studies on exosome biology and function, and for the development of exosome-based biomarkers and therapeutics.

## Results

### Cells release distinct subpopulations of exosomes

**Figure 1:**
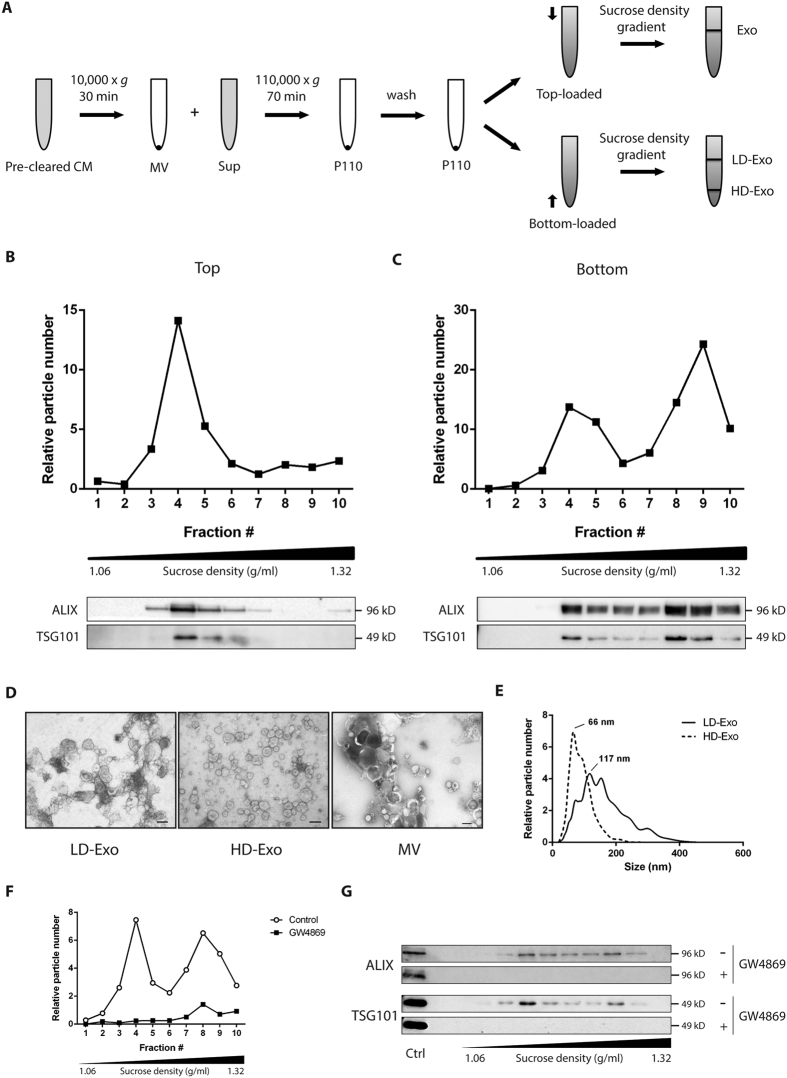
B16F10 melanoma cells release distinct subpopulations of exosomes. (**A**) Schematic overview of the isolation protocol used to obtain different exosome populations. (**B,C**) P110 was loaded on top (**B**) or at the bottom (**C**) of a sucrose density gradient and subjected to ultracentrifugation for 16 h. The resulting fractions (1–10) with increasing density were analyzed for particle number by NTA (upper panels) and the presence of exosome marker proteins ALIX and TSG101 by Western blotting (lower panels). For Western blots, an equal volume of each sample was analyzed. Fractions 3–5 (LD-Exo) and 8–9 (HD-Exo) were pooled for further analysis. (**D**) Exosomes or MVs were negatively stained with uranyl acetate and visualized by transmission electron microscopy. Scale bars represent 100 nm. (**E**) Size distribution profiles as determined by NTA. Data shown are representative of three independent experiments. (**F,G**) Exosomes from B16F10 melanoma cells cultured for 48h in the absence or presence of 7.5 μM GW4869 were isolated as in Fig. 1A. P110 was loaded at the bottom of a sucrose gradient and subjected to ultracentrifugation for 16 h. The resulting fractions (1–10) with increasing density were analyzed for particle number by NTA (**F**) and the presence of exosome marker proteins ALIX and TSG101 by Western blotting (**G**). A control sample (Ctrl) was included to show consistent analysis across the membranes. For Western blots, an equal volume of each sample was analyzed. Data shown are representative of two independent experiments.

For isolation of MVs and exosomes, conditioned medium (Opti-MEM) from B16F10 melanoma cells was collected, pre-cleared of cells and debris and subjected to a series of (ultra)centrifugation steps (Fig. 1A). The resulting 110,000 × g pellet (P110), usually referred to as the exosome pellet, was subsequently subjected to sucrose density gradient centrifugation. Others have previously suggested that sucrose density gradients allow separation of exosome subpopulations^20^,^22^,^23^. When we loaded P110 on top of the sucrose gradient followed by ultracentrifugation for 16 h, we detected the vast majority of particles, as well as the exosome marker proteins ALIX and TSG101, in fractions 3–5 (Fig. 1B). These fractions correspond to a density range of 1.12–1.19 g/ml, which is in accordance with the originally proposed density of exosomes^9^. By contrast, when P110 was loaded on the bottom and allowed to float into the gradient, two distinct particle populations could be distinguished, in both of which ALIX and TSG101 were detected (Fig. 1C). One population was again recovered from the lower density fractions 3–5 (LD-Exo), while the second population associated with the higher density fractions 8–9 (HD-Exo), with densities of 1.26–1.29 g/ml. ALIX was found to be enriched in both LD-Exo and HD-Exo compared to cell lysate and to MVs, while the opposite was observed for the endoplasmic reticulum protein Calnexin (Supplementary Fig. S1A). Furthermore, proteomic analysis (see below) showed enrichment of the transmembrane tetraspanin proteins CD63, CD9 and CD81 in both LD- and HD-Exo as compared to MVs (Supplementary Fig. S1B). The two populations were also obtained when we used medium with exosome-depleted FCS instead of Opti-MEM as exosome isolation medium (Supplementary Fig. S2A).

We then also subjected P110 to sucrose density gradient centrifugation for 72 h. Regardless of whether P110 was loaded on top of the gradient or on the bottom, particles as well as ALIX and TSG101 were mainly detected in fractions 3–5, which suggests that both populations equilibrate at the same density of 1.12–1.19 g/ml, but HD-Exo display delayed flotation behavior (Supplementary Fig. S3A)^22^.

To rule out the possibility that subpopulations of exosomes were an artifact of the ultracentrifugation method, which has been shown to affect exosome integrity, we also employed ultrafiltration followed by size-exclusion chromatography (UF-SEC) for exosome isolation. This method, in contrast to ultracentrifugation, allows for recovery of exosomes with preserved biophysical properties^24^. When we allowed UF-SEC-purified exosomes to float into a sucrose gradient, again two distinct subpopulations could be recovered (Supplementary Fig. S2B). In addition, to verify that this delayed flotation behavior was not caused by osmotic damage to the exosomes as a consequence of the hyperosmotic nature of sucrose gradients, we subjected P110 to Nycodenz density gradient centrifugation. Similarly to sucrose, Nycodenz is a nonionic gradient-forming material, however the osmolarity of Nycodenz solutions is considerably lower than that of sucrose. Importantly, also using a Nycodenz gradient, two exosome subpopulations could clearly be distinguished (Supplementary Fig. S2C).

Transmission electron microscopy analysis indicated that both LD-Exo and HD-Exo are spherical, membrane-encapsulated particles with a cup-shaped morphology, characteristic of exosomes (Fig. 1D). In contrast, large membrane vesicles, pelleted at 10,000 × g (MVs), represent a heterogeneous population of EVs, differing greatly in size, shape and electron-density. The majority of LD-Exo were in a size range of 75–200 nm, while HD-Exo were smaller and more homogeneous in size, with most vesicles smaller than 100 nm. This finding was confirmed by NTA, which showed distinct size distribution profiles for the LD-Exo and HD-Exo populations, with LD-Exo peaking at 117 nm and HD-Exo peaking at 66 nm (Fig. 1E).

Budding of exosome vesicles has been shown to be dependent on the conversion of sphingomyelin into ceramide by neutral sphingomyelinase (nSMase)^14^. To evaluate the role of nSMase in the formation of both subpopulations of exosomes, we treated cells with the nSMase inhibitor GW4869. Based on the number of particles (Fig. 1F) and the expression levels of ALIX and TSG101 (Fig. 1G), exosome release was almost completely inhibited after treatment with GW4869. This indicates that both exosome subpopulations are formed through a ceramide-dependent pathway.

**Figure 2:**
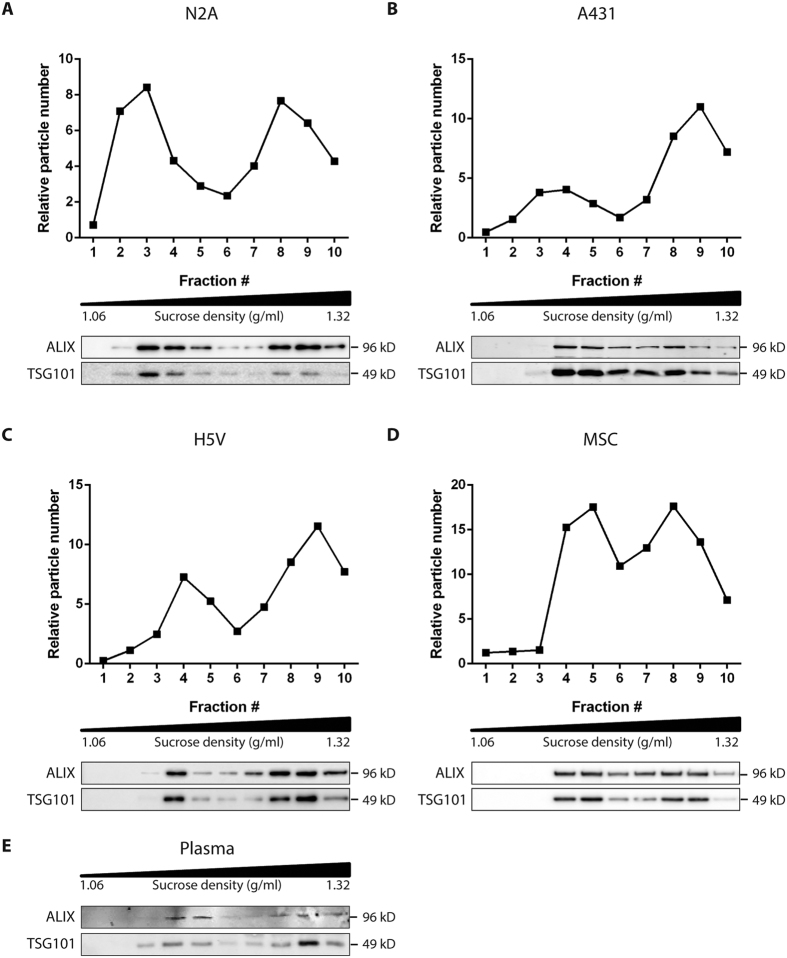
Exosome subpopulations are released by different cell types and are detected in plasma. Exosomes derived from (**A**) N2a cells, (**B**) A431 cells, (**C**) H5V cells, (**D**) MSC cells, or (**E**) plasma were isolated as in Fig. 1A. P110 was loaded at the bottom of a sucrose density gradient and ultracentrifugated for 16 h. The resulting fractions (1–10) with increasing density were analyzed for particle number by NTA (upper panels) and the presence of exosome marker proteins ALIX and TSG101 by Western blotting (lower panels). For Western blots, an equal volume of each sample was analyzed. Data shown are representative of two independent experiments.

To test whether release of such distinct exosome subpopulations is exclusive to melanoma cells, we also isolated exosomes from other cell sources (i.e. N2a mouse neuroblastoma cells, A431 human squamous carcinoma cells, H5V mouse heart endothelial cells, MSC hTERT immortalized mesenchymal stem cells), and plasma, and evaluated the presence of LD-Exo and HD-Exo subpopulations on a sucrose density gradient. Although subtle differences in flotation behavior among exosomes from different cell sources were observed, two distinct subpopulations of exosomes could be clearly distinguished in all exosome samples studied (Fig. 2). Together, these data demonstrate that cells release distinct subpopulations of exosomes which can be separated using sucrose density gradient centrifugation.

### Exosome subpopulations have distinct protein compositions

**Figure 3:**
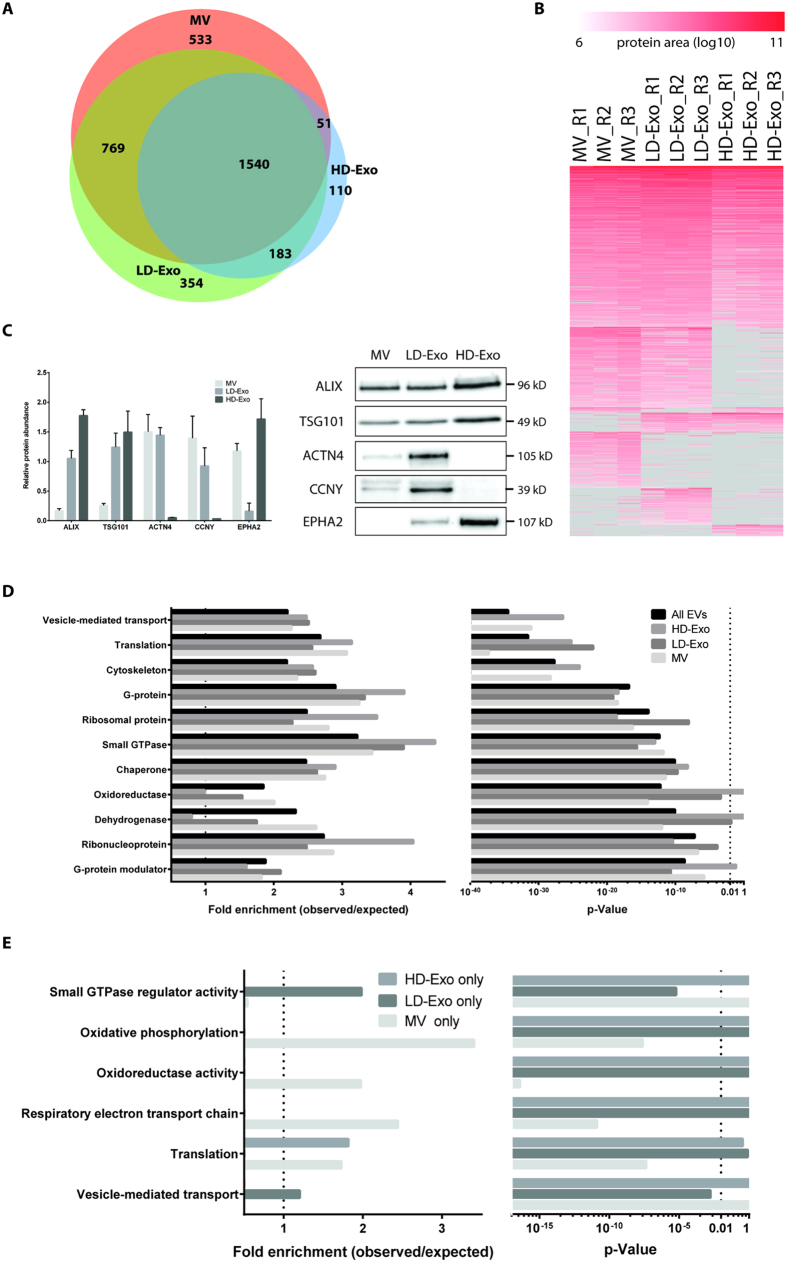
Proteome characterization of EV subpopulations by nanoLC-MS/MS reveals differences in protein composition. (**A**) Overlap of identifications between MV, LD-Exo and HD-Exo. Proteins were considered identified if they had quantitative protein area in at least 2 of the 3 replicates. (**B**) Heatmap showing protein intensity (area) for each replicate. Subpopulations were grouped based on overlap in identifications as in (**A**), and subsequently ranked from highest to lowest average protein area. (**C**) Validation of proteomics data for selected proteins (left panel) by Western blotting (right panel). Equal amounts of protein were analyzed. (**D**) GO enrichments of all identified proteins and proteins identified in MV, LD-Exo and HD-Exo. (**E**) GO enrichments of proteins uniquely identified in MV, LD-Exo and HD-Exo.

To further characterize the distinct exosome subpopulations, we investigated their protein composition using label-free mass spectrometry-based proteomics. Analysis was performed in triplicate for LD-Exo and HD-Exo, and for larger membrane vesicles pelleted at 10,000 × g (MV) for comparison. In total, we identified 4421 proteins (1% FDR) from all EVs with good overlap in protein identification between replicates, and a median number of peptide spectrum matches (psms)/protein between 4 and 7 (Supplementary Table S1, Supplementary Fig. S4A–D). A more conservative filtering was applied in the following analysis, where proteins were considered detected if they were identified (1% FDR) and quantified (had protein quantitative information, protein area defined) in at least 2 of the 3 replicates. Using this criterion, 1540 proteins were identified in all vesicle populations, and 533, 354 and 110 proteins were identified exclusively in MV, LD-Exo and HD-Exo, respectively. 769 proteins were identified in both MV and LD-Exo, while 183 proteins were identified in both LD-Exo and HD-Exo, and 51 proteins in both MV and HD-Exo (Fig. 3A,B). Protein quantitation correlated well between replicates (Supplementary Fig. S5A-C). Proteins common to all populations included suggested EV and exosome marker proteins ALIX, TSG101, CD9, CD81 and CD63, although their relative abundance was higher in LD-Exo and HD-Exo as compared to MV. As orthogonal confirmation of protein enrichment to LD-Exo and HD-Exo detected by MS-analysis, selected proteins were analyzed by Western blotting. As in MS-analysis, actinin alpha 4 (ACTN4) and cyclin Y (CCNY), were enriched in LD-Exo versus HD-Exo, and of ephrin type-A receptor 2 (EPHA2) was enriched in HD-Exo versus LD-Exo (Fig. 3C). After sucrose density gradient centrifugation for 72 h, EPHA2 was mainly detected in fractions 3–5 (LD fractions), confirming that EPHA2 is associated with exosomes that display delayed flotation behavior (HD-Exo), but eventually equilibrate at a density of 1.12–1.19 g/ml (Supplementary Fig. S3B). We also loaded P110 on a size exclusion column packed with Sephacryl S-1000, and separated the exosomes into two fractions (Supplementary Fig. S6A). Fraction 1, which contained larger exosomes, was found to be enriched for ACTN4 and CCNY, while fraction 2, which contained smaller exosomes, was enriched for EPHA2 (Supplementary Fig. S6B,C). This further indicates that these proteins are indeed selectively enriched in large or small exosomes, respectively. Selective enrichment of ACTN4 and CCNY in LD-Exo was also found for exosomes isolated from H5V cells (EPHA2 could not be detected in H5V exosomes) (Supplementary Fig. S7).

To understand the potential functional differences between the proteins identified in the distinct vesicle subpopulations, we performed GO enrichment analysis using 4 different protein lists: (1) the full list of all identified proteins across EV populations, and (2) MV, (3) LD-Exo and (4) HD-Exo protein identifications, compared to the whole proteome database (Fig. 3D). Commonly enriched terms in all 3 subpopulations included ‘vesicle mediated transport’, ‘translation’, ‘cytoskeleton’, ‘G-protein’, ‘ribosomal protein’, ‘small GTPase’ and ‘chaperones’, aligning our previous observations on exosome content^24^,^25^. ‘Oxidoreductases’ and their subgroup ‘dehydrogenases’ were enriched in MV, and slightly enriched in LD-Exo but not in HD-Exo, potentially reflecting the presence of mitochondrial components in MV and LD-Exo. The terms ‘translation’, ‘ribonucleoproteins’ and ‘ribosomal proteins’ displayed higher enrichment in HD-Exo than in LD-Exo. ‘G-proteins’ were enriched in both HD-Exo and LD-Exo, while only LD-Exo reached significant enrichment for ‘G-protein modulators‘. For proteins considered uniquely identified in one of the EV subpopulations (Fig. 3A), LD-Exo showed enrichment in ‘small GTPase regulatory activity’ and ‘vesicle mediated transport’. HD-Exo unique proteins did not show any enrichment in a GO-specific category. In contrast, MV unique proteins displayed enrichment in ‘oxidoreductase’, ‘oxidative phosphorylation’ and ‘respiratory electron transport chain’ (Fig. 3E). Thus, LD-Exo and HD-Exo represent two distinct subpopulations of exosomes with unique protein compositions, both differing from larger MVs.

### Exosome subpopulations have distinct RNA profiles

**Figure 4:**
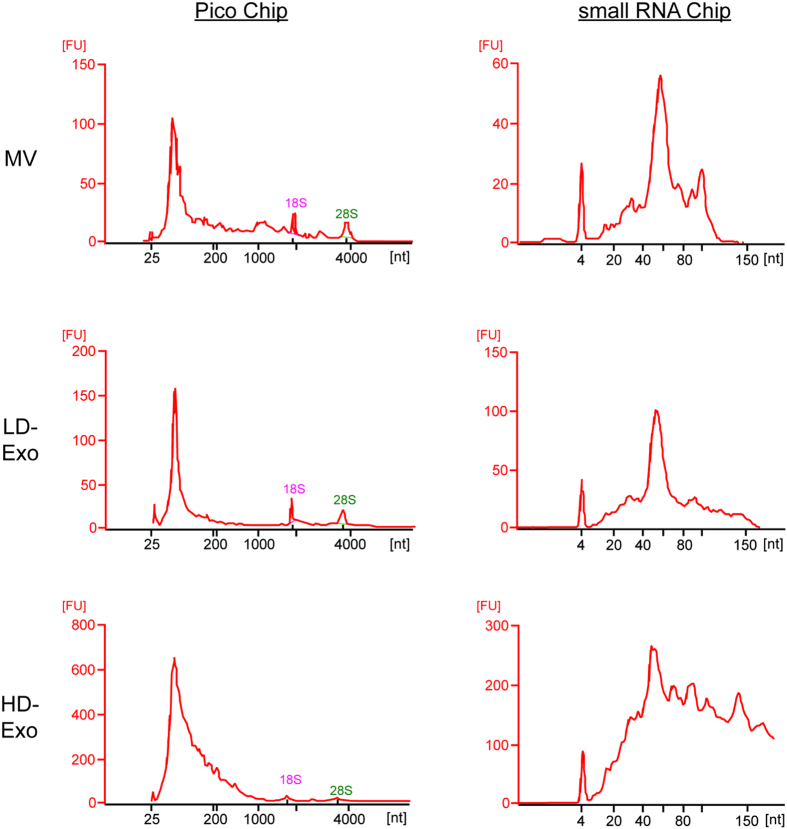
EV subpopulations have different RNA profiles. RNA from MV, LD-Exo and HD-Exo was extracted using Trizol and analyzed using capillary electrophoresis with the Agilent RNA 6000 Pico chip (left panel) and Agilent small RNA chip (right panel) on an Agilent 2100 Bioanalyzer®. The y-axis of the electropherograms represents fluorescence units (FU) and the x-axis represents the nucleotide length of the RNA (nt). Peaks at 25 nt (left panels) or at 4 nt (right panels) represent internal standards. Data shown are representative of two independent experiments.

EVs contain different RNA species, including mRNA (and fragments thereof), long and small non-coding RNA and ribosomal RNA (rRNA). However, the relative amounts of different species of RNA vary depending on the EV source and on the methodology used to isolate EVs and to obtain the data^11^. To explore the differences in RNA size distribution profiles of MV, LD-Exo and HD-Exo, we isolated total RNA from each source and analyzed it by Bioanalyzer^®^ (Fig. 4). In general, the RNA profiles of MV and LD-Exo were very similar. When run on an Agilent RNA 6000 Pico chip, three dominant peaks were observed for MV and LD-Exo, two of which corresponded to rRNA subunits 18S and 28S. The third peak was found at ~60 nt. In contrast, HD-Exo did not contain rRNA, and a broader RNA size distribution profile was observed in the range of 50–200 nt. On an Agilent RNA small RNA chip, again one dominant peak was observed at ~60 nt for MV and LD-Exo, while for HD-Exo, RNA sizes ranged from 30–150 nt. This indicates that LD-Exo and HD-Exo have a distinct RNA content.

### Distinct exosome subpopulations differentially affect gene expression in recipient cells

**Figure 5:**
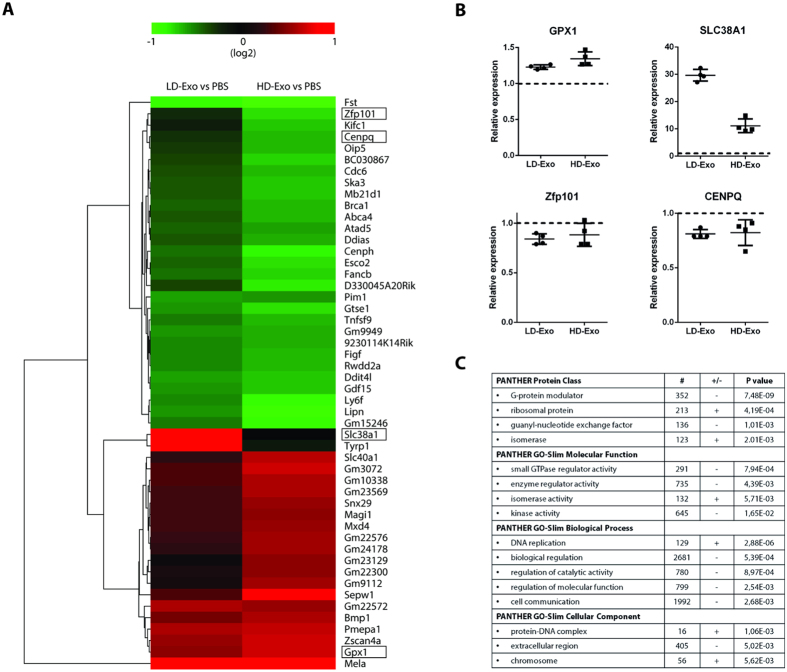
LD-Exo and HD-Exo have different effects on gene expression in recipient cells. LD-Exo and HD-Exo were isolated as in Fig. 1A and incubated with H5V endothelial cells for 24 h. (**A**) Heat map displaying genes that were upregulated (red) or downregulated (green) in response to LD-Exo or HD-Exo exposure (>1.5 fold change, q-value < 0.15). (**B**) RT-qPCR validation of array results. Transcript levels were measured relative to GAPDH and plotted relative to levels in PBS-treated cells. Dot plots represent mean ± SD. (n = 4). (**C**) Representative GO enrichments differentially regulated (upregulated,  + , or downregulated, -) by LD-Exo versus HD-Exo. #, number of genes.

Since LD- Exo and HD-Exo are biophysically distinct and have different protein and RNA compositions, we next evaluated whether these exosome subpopulations might also be functionally distinct. To address this, LD-Exo and HD-Exo were incubated with H5V endothelial cells for 24 h, and changes in gene expression in exosome-recipient cells were evaluated using a Genechip gene array. Overall, 257 and 1116 genes were found to be differentially expressed in endothelial cells treated with LD-Exo and HD-Exo, respectively, compared to control PBS-treated cells (Supplementary Table S2) (q-value < 0.15). Out of these, 42 genes had a greater than 1.5-fold expression change upon exposure to either LD-Exo or HD-Exo (11 genes upregulated, 31 genes downregulated) (Fig. 5A). We next validated the results obtained from the gene array using RT-qPCR, which confirmed upregulation of glutathione peroxidase 1 (GPX1) and downregulation of zinc finger protein 101 (Zfp101) and centromere protein Q (CENPQ) by both exosome types, and a much more pronounced upregulation of solute carrier family 38, member 1 (SLC38A1) by LD-Exo compared to HD-Exo (Fig. 5B). “Empty” sucrose fractions 3–5 and 8–9 (prepared by floating non-conditioned OptiMEM) had no effect on gene expression (Supplementary Fig. S8). In order to understand the more global effects elicited by these two distinct exosome subpopulations, we performed GO statistical enrichment analysis on the genes affected by LD-Exo and HD-Exo, and compared the results. Several gene classes were found to be differentially affected by the exosome subpopulations, most significantly PANTHER protein classes ‘G-protein modulator’ and ‘ribosomal protein’, GO molecular function ‘small GTPase regulator activity’ and ‘enzyme regulator activity’, GO biological process ‘DNA replication’ and ‘biological regulation’ and GO cellular component ‘protein-DNA complex’ and ‘extracellular region’ (Fig. 5C, Supplementary Table S3). These results suggest that distinct subpopulations of exosomes differentially affect gene expression and therefore have distinct biological effects on recipient cells.

## Discussion

Throughout the EV field, exosomes are assumed to represent a homogenous population of EVs. Previous research has mainly focussed on comparative analysis of classic EV subtypes, i.e. apoptotic bodies, microvesicles and exosomes^26^,^27^,^28^,^29^, and of EVs being released from the apical and basolateral surfaces of organoids^30^. Here, we reveal heterogeneity within exosomes, demonstrating that cells release at least two major subpopulations of exosomes with distinct molecular compositions and biological properties, and this finding is consistent across several cell types as well as for plasma.

Exosome subpopulations were detected based on a difference in migration speed in a sucrose gradient, a method previously used by others to study the heterogeneity of exosomes^20^,^22^,^23^. We used an elaborate isolation procedure (Fig. 1A) to maximize the purity of the exosome preparations, and performed subsequent experiments with exosomes pooled from fractions 3–5 (LD-Exo) and 8–9 (HD-Exo) from a sucrose density gradient (Fig. 1C), to minimize mutual contamination. Both populations expressed the exosome marker proteins ALIX and TSG101, displayed the typical cup-shaped morphology in electron microscopy preparations and were formed through a ceramide-dependent pathway. Furthermore, both populations equilibrated at the same originally proposed density of exosomes of 1.12–1.19 g/ml. Together, these lines of evidence strongly suggest that both populations are indeed exosomes. The differences in migration speed in the sucrose gradient could be explained by the different particle sizes for each subpopulation (LD-Exo: mode size 117 nm, HD-Exo: mode size 66 nm, Fig. 1E), as besides density, the rate at which particles migrate through a sucrose gradient is also dependent on their size.

Proteomic analysis revealed common as well as unique proteins among the different EV types. Among the proteins that were found in MVs as well as both exosome subpopulations isolated from melanoma cells, we found suggested EV and exosome marker proteins ALIX, TSG101, CD9, CD81 and CD63, however their relative abundance was higher in LD-Exo and HD-Exo as compared to MVs. The presence of exosome marker proteins in the MV samples could indicate that a fraction of exosomes is co-purified at 10,000 × g, or that these proteins cannot be used to distinguish exosomes from larger membrane vesicles. Interestingly, when comparing the two exosome subpopulations, substantial differences in composition were found. We confirmed selective enrichment of ACTN4 and CCNY in LD-Exo versus HD-Exo (Fig. 3C). Selective enrichment of ACTN4 and CCNY on LD-Exo was also detected for exosomes isolated from other cell lines (i.e. H5V), suggesting that these proteins could be used as marker proteins specifically for larger exosomes. CCNY is a highly conserved membrane associated cyclin, and part of the cyclin superfamily of proteins which play a crucial role in cell cycle regulation and transcription^31^. ACTN4 is a cytoskeleton-associated protein, previously shown to be involved in metastasis of cancer^32^, and has also been identified as a potential biomarker for cervical cancer^33^. On the other hand, EPHA2 was found to be enriched in HD-Exo versus LD-Exo. Eph-Ephrin interactions are involved in numerous cellular processes, such as regulation of vascular endothelial growth factor (VEGF) signalling and angiogenesis^34^. As a role for exosomes in cancer spread is well established^7^, enrichment of specific proteins such as EPHA2 could indicate the potential involvement of unique exosome subpopulations in different cellular processes related to oncogenesis.

Analysis of GO enrichments across the three populations provided insight to the potential functional differences between the identified proteins, and further highlighted the unique protein composition of LD-Exo and HD-Exo. ‘Vesicle mediated transport’, ‘translation’, ‘cytoskeleton’, ‘G-protein’, ‘ribosomal protein’, ‘small GTPase’ and ‘chaperones’, were commonly enriched terms, a finding which is in line with our previous observations on exosome content (15, 17). GO terms ‘translation’, ‘ribonucleoproteins’ and ‘ribosomal proteins’ displayed higher enrichment in HD-Exo than in LD-Exo. Interestingly, we only found 18S and 28S ribosomal RNA in MVs and LD-Exo, while HD-Exo contained a broad range of RNA species ranging in size from 30 – 150 nt. rRNA has previously been found in MVs^35^, and also in exosomes^36^, although others have reported that exosomes are negative for intact 18S and 28S rRNA^6^,^35^,^37^. This apparent discrepancy could be explained by differences in the exosome isolation protocol used (resulting in more or less co-purification of MVs), differences in cell type, or, as our data point towards, the presence of distinct exosome subpopulations.

GO enrichment analysis did not provide direct evidence regarding differences in biogenesis of the subpopulations. Inhibition of cellular ceramide formation by inhibition of neutral sphingomyelinase (nSMase) activity however resulted in a substantially decreased release of both populations (Fig. 1F,G). This indicates that both exosome subpopulations are formed through a ceramide-dependent pathway, which may suggest both subpopulations are of endosomal origin, although the role of ceramides in the formation of other types of EVs remains to be determined^14^. It has previously been shown that cells contain different populations of multivesicular bodies (MVBs), that can contain both large as well as small intraluminal vesicles (ILVs)^9^,^13^. ILV size is influenced by the ILV cargo and its mechanism of formation, which comprise ESCRT-dependent and –independent processes operating within the same cell and/or MVB. Based on our findings, it is tempting to speculate that LD-Exo and HD-Exo are derived from different types of ILVs, but whether their size difference is a result of their cargo or of the activation of different mechanisms controlling their formation remains to be investigated.

It has been well-established that exosomes play a role in intercellular communication through the transfer of their cargo^1^,^11^. We evaluated potential functional differences by studying the effects of melanoma-derived LD-Exo and HD-Exo on gene expression in recipient endothelial cells (Fig. 5). For LD-Exo, 257 genes were significantly changed versus control cells, while for HD-Exo, 1116 genes were significantly altered. Further analysis of 42 genes with a greater than 1.5-fold expression change revealed an upregulation of 11 genes and a downregulation of 31 genes. Our findings highlight the diverse biological effects that EVs can mediate. We observed a much more pronounced upregulation of SLC38A1 by LD-Exo compared to HD-Exo. SLC38A1 is involved in membrane transport of glutamine, and cancerous cells show enhanced uptake of glutamine. Interestingly, others have shown that glutamine metabolism was altered in cancer cells exposed to large EVs, but not exosomes^29^.

Although the differences in regulation of individual genes between the two subpopulations were found to be limited, GO statistical enrichment analysis revealed that a number of protein classes, molecular functions, biological processes and cellular components were differentially regulated by LD-Exo versus HD-Exo. Whether subpopulations of exosomes also display differences in regulation on a protein level, have distinct effects on cellular activation status and show different cell uptake and/or targeting behavior requires further investigation.

In conclusion, our results clearly show that cells secrete distinct populations of exosomes with unique size, protein and RNA composition, and that these exosomes have different effects on recipient cells. Discrimination between subpopulations of exosomes will therefore be of great importance for studies on exosome biology and function, and assist in the development of exosome-based diagnostics and therapeutics.

## Methods

### Cell culture

B16F10 cells (ATCC, Manassas, VA, USA), A431 cells (ATCC), N2a (ATCC) and H5V cells (ATCC) were cultivated in DMEM (Life Technologies, Carlsbad, CA, USA), hTERT immortalized mesenchymal stem cells were kindly provided by St. Jude Children’s Research Hospital, and were cultivated in RPMI (Life Technologies). Media were supplemented with 10% heat-inactivated FCS (Life Technologies), 100 U/ml penicillin (Sigma-Aldrich, St. Louis, MO, USA) and 250 ng/ml amphotericin B (Sigma). Cells were cultivated at 37 °C with 5% CO_2_. Cells were routinely checked for mycoplasma contamination using the PCR Mycoplasma Test Kit I/C (Promokine, Heidelberg, Germany).

### EV isolation by differential ultracentrifugation

B16F10 cells were seeded in 150 mm^2^ polystyrene dishes (Corning, NY, USA). After 24 h, cells were washed with PBS and medium was replaced for 20 mL Opti-MEM (Life Technologies) or 20 mL DMEM (Life Technologies) supplemented with 10% FCS depleted of EVs. EVs were removed from FCS by overnight centrifugation at 110,000 × g. Conditioned medium (CM) was collected 48 h after media exchange when cells had reached 90–100% confluency (approximately 20 × 10^6^ cells per 150 mm^2^ dish). CM was spun at 2000 × g for 10 minutes to remove cells and cell debris. Microvesicles (MV) were isolated by centrifugation at 10,000 × g for 30 minutes, and subsequently washed by resuspending the pellet in 25 ml of PBS (Life Technologies) and centrifugation at 10,000 × g for 30 minutes. Exosomes were isolated by centrifugation of the 10,000 × g supernatant for 70 minutes at 110,000 × g, and subsequently washed by resuspending the pellet in 25 ml of PBS (Life Technologies) and centrifugation at 110,000 × g for 70 minutes. For EV isolation from plasma, whole blood from healthy volunteers was collected in vacuum tubes containing trisodium citrate (final concentration 0.32% (w/v)) and centrifuged twice at 2000 × g for 10 min at room temperature (RT) to generate platelet-free plasma. Procedures were approved by the University Medical Center Utrecht Ethical Committee. Plasma was diluted six times in PBS, and MVs and exosomes were isolated as above. All ultracentrifugation steps were performed using the Beckman Coulter (Brea, CA, USA) Type 55.2 Ti rotor (k factor 64) at 4 °C.

### EV isolation by size-exclusion liquid chromatography

Cells were handled as described under ‘EV isolation with differential ultracentrifugation’. Exosomes were isolated from ~250 mL conditioned medium as previously described^24^. Briefly, the 10,000 × g supernatant was concentrated with 100 kDa molecular weight cut-off Amicon centrifugal filter units (Merck Millipore, Billerica, Massachusetts, USA) to 1 ml and subsequently loaded on a HiPrep Sephacryl S-400 HR 16/60 size exclusion chromatography column (GE Healthcare, Buckinghamshire, UK) connected to the ÄKTA prime system (GE Healthcare), and eluted at 0.5 ml/min flow rate using PBS as the eluent. Chromatogram was recorded using absorbance at 280 nm. 2 ml fractions were collected, and EV-containing fractions were pooled and concentrated with 10 kDa molecular weight cut-off Amicon centrifugal filter units (Merck Millipore).

### Sucrose density gradient

Exosomes were either covered with ( = bottom loading/upward displacement) or loaded on top of a discontinuous sucrose step-gradient ranging from 0.4–2.5M sucrose (Sigma) in HEPES buffer pH 7.4 (Sigma), and centrifuged at 200,000 × g for 16 hours (unless otherwise indicated) in a SW41 swinging bucket (Beckman) at 4 °C. After centrifugation, 1 ml fractions were collected and each fraction was weighed to determine its average density. The low density exosome subpopulation (LD-Exo) was obtained by pooling sucrose fractions 3–5, while the high density subpopulation (HD-Exo) was obtained by pooling sucrose fractions 8–9. Pooled fractions were diluted in PBS and placed on a SSL4 See-saw rocker (Stuart, Staffordshire, UK) at 4 °C for 24 h. Exosomes were collected by centrifugation at 110,000 × g for 70 minutes.

### Nanoparticle tracking analysis

Nanoparticle tracking analysis (NTA) was performed with a NS500 nanoparticle analyser (NanoSight, Malvern, Worchestershire, UK). Camera level was set at 14 for all recordings. Camera focus was adjusted to make the particles appear as sharp individual dots. Three 30-second videos were recorded for each sample with a delay of 7 seconds between each recording. All post-acquisition functions were set at automatic, except detection threshold, which was set at 4.

### SDS-PAGE and Western blot analysis

Samples were resuspended in Laemmli sample buffer (Bio-Rad, Hercules, CA, USA) with freshly added β-mercaptoethanol (5% (v/v)) (Sigma). Samples were subsequently heated for 10 minutes at 95 °C and separated on a 1.5 mm, 10% SDS (Sigma) polyacrylamide gel alongside a molecular weight marker (Rainbow Protein Marker, Coloured, GE Healthcare). Blotting was performed on an Immobilin®-FL PVDF membrane (Merck Millipore). Membranes were blocked in 5% skimmed milk in TBS containing 0.1% Tween-20 (TBS-T) for 1 h, primary antibodies (mouse anti-ALIX (Abcam, ab117600, Cambridge, UK) at 1:1000, rabbit anti-TSG101 (Abcam, ab30871) at 1:1000, rabbit anti-alpha actinin 4 (Abcam, ab137564) at 1:500, rabbit anti-cyclin Y (Abcam, ab114186) at 1:500 and rabbit anti-Eph receptor A2 (Abcam, ab5387) at 1:1000) were incubated overnight in 5% skimmed milk in TBS-T at 4 °C. After incubation, membranes were washed three times in TBS-T for 10 minutes at RT. Membranes were subsequently probed with IRDye® 680RD Goat anti-Rabbit (Li-COR, Nebraska, USA) or IRDye® 800CW Goat anti-Mouse (Li-COR) at 1:10,000 in TBS-T with 0.01% SDS for 2 hours at RT. After incubation, membranes were washed three times in TBS-T for 10 minutes at RT and imaged on an Odyssey FC Imager (Li-COR).

### NanoLC-MS/MS Proteomic analysis

MVs and exosomes were isolated from ~1000 mL conditioned medium. Purified EVs were lysed with 1% SDS, 25 mM HEPES, 1 mM DTT. Lysates were heated to 95 °C for 5 min followed by sonication for 1 min and centrifugation at 14,000 g for 15 min. The supernatant was mixed with 1 mM DTT, 8 M urea, 25 mM HEPES, pH 7.6 and transferred to a 10-kDa cut-off centrifugation filtering unit (Pall, Nanosep®, NY, USA), and centrifuged at 14,000g for 15 min. Proteins were alkylated by 50 mM iodoacetamide (IAA) in 8 M urea, 25 mM HEPES for 10 min. The protein lysates were then centrifuged at 14,000g for 15 min followed by 2 more additions and centrifugations with 8 M urea, 25 mM HEPES. Trypsin (Promega) in 250 mM urea, 50 mM HEPES was added to the cell lysate at a ratio of 1:50 trypsin:protein and incubated overnight at 37 °C. The filter units were centrifuged at 14,000g for 15 min followed by another centrifugation with MQ and the flow-through was collected. Peptides were cleaned by a strata-X-C-cartridge (Phenomenex).

Before analysis on the Q Exactive (Thermo Fischer Scientific, San Jose, CA, USA), peptides were separated using an Ultimate 3000 RSLCnano system. Samples were trapped on an Acclaim PepMap nanotrap column (C18, 3 μm, 100 Å, 75 μm x 20 mm), and separated on an Acclaim PepMap RSLC column (C18, 2 μm, 100Å, 75 μm x 50 cm), (Thermo Scientific). Peptides were separated using a gradient of A (5% DMSO, 0.1% FA) and B (90% ACN, 5% DMSO, 0.1% FA), ranging from 6% to 37% B in 240 min with a flow of 0.25 μl/min. The Q Exactive was operated in a data dependent manner, selecting top 10 precursors for fragmentation by HCD. The survey scan was performed at 70.000 resolution from 400–1600 m/z, with a max injection time of 100 ms and target of 1 × 106 ions. For generation of HCD fragmentation spectra, a max ion injection time of 140 ms and AGC of 1 × 105 were used before fragmentation at 30% normalized collision energy, 35,000 resolution. Precursors were isolated with a width of 2 m/z and put on the exclusion list for 70 s. Single and unassigned charge states were rejected from precursor selection.

### Peptide, protein identification and data analysis

Proteome discoverer 1.4 with SequestHT was used for protein identification. Precursor mass tolerance was set to 10 ppm and for fragments to 0.02 Da. Oxidized methionine was set as dynamic modification, and carbamidomethylation as static modification. Spectra were matched to a Mus musculus, Ensembl 75 database, supplemented with the 250 most abundant Bos taurus proteins from FCS, and results were filtered to 1% FDR. Identifications in Bos taurus were considered to originate from FCS and removed. Protein areas were normalized to the median between each replicate to account for uneven peptide loading. Proteins were considered identified if they had quantifiable protein area in 2 or more of the 3 biological replicates. GO term enrichment analysis was done using Panther^38^.

### Transmission Electron Microscopy

10 μl of MV or exosome suspension was added onto formvar-carbon coated electron microscopy grids (Agar Scientific, Elektron Technology UK Ltd, Essex, UK) for 20 min. The grid was blotted dry with filter paper and 15 μL of 2% uranyl acetate Sigma) was added on the grid for 1 minute. Next, uranyl acetate was removed and 15 μL of distilled water was added for 1 minute. The water droplet was then removed and the grid was left to air dry for 15 minutes. The grids were then visualized using a transmission electron microscope (JEM-1010, JEOL Ltd, Tokyo, Japan).

### RNA extraction and profiling

MVs and exosomes were isolated from ~250 mL conditioned medium. RNA was extracted using TRIzol®-LS Reagent (Life Technologies) according to manufacturer’s instructions. Isolated RNA was resuspended in RNase free water and RNA yield was determined with Quant-iT™ RiboGreen® RNA assay (Life Technologies) according to the manufacturer’s protocol. Quality and size of RNA in exosomes were determined using capillary electrophoresis with the Agilent RNA 6000 Pico kit and Agilent RNA small RNA kit on an Agilent 2100 Bioanalyzer® (Agilent Technologies, Santa Clara, CA, USA) according to the manufacturer’s protocol.

### Gene array

H5V cells were seeded in 24 well plates at a density of 60,000 cells per well in DMEM (Life Technologies) supplemented with 10% HI FCS depleted of serum EVs. After 24 h, cells were incubated with LD-Exo or HD-Exo isolated from ~500 mL conditioned medium, at a concentration of 15 μg/ml (or 30 μg/ml for RT-qPCR validation) or PBS for 24 h. Cells were washed three times with PBS, and RNA was isolated using RNeasy Mini Kit (Qiagen, Hilden, Germany) according to the manufacturer’s protocol, Isolated RNA was resuspended in Nuclease free water (Life Technologies) and RNA yield was determined using the NanoDrop 2000 UV-Vis Spectrophotometer (Thermo Scientific). The RNA quality was assessed using Agilent 2100 Bioanalyzer® (Agilent Technologies, Santa Clara, CA, USA). 100 ng of total RNA was run for each sample on Mouse Gene ST 2.1 Array plates (Affymetrix, Santa Clara, CA, USA). Labeling and hybridisation were performed according to standard Affymetrix protocols at the Bioinformatics and Expression Analysis (BEA) core facility, at Novum, Huddinge, Sweden. The processing and data analyses were performed in Affymetrix Expression Console Software. The arrays were analysed using Median Polish, RMA background correction and Sketch-Quantile normalisation (RMA-Sketch in Expression Console Software). Only the signals from the probe sets in the main category were used. If a probe set signal was below 20 in all the groups, it was removed. False Discovery Rate (FDR) estimations were done calculating q values (qvalue R package) from the p-value lists. The criterion for differentially expressed transcripts was set to q < 0.15. The microarray data are accessible through GEO (GSE72351).

To analyse whether certain gene ontology (GO) terms were enriched among the genes that were differentially regulated by LD-Exo and HD-Exo treatment, we used the PANTHER Gene Ontology Classification System tool^38^. We calculated fold changes in gene expression between samples treated with LD-Exo and HD-Exo, and subjected these data to statistical enrichment analysis using the Mann-Whitney test^39^. The Mann-Whitney test is a rank-sum test that compares whether the distribution of LD-Exo/HD-Exo fold-change values of a given GO term is statistically different to the distribution of all fold-change values in the data set. If the genes of a given GO term are sampled in a statistically significant manner from among the higher ranking genes of the distribution of all fold-change values, the given GO term is said to be “statistically enriched/upregulated” ( + ) in the dataset. Accordingly, vice versa, when genes of the GO term are sampled from among the lower ranking genes, the GO term is said to be “statistically de-enriched/downregulated” (-) in the dataset. The results include also the number of genes (#) belonging to those GO terms. Note that the Mann-Whitney test compares the distributions of gene expression fold-changes, and not the expression level of individual genes separately.

For selected genes, changes were validated with RT-qPCR. cDNA was synthesized from 200 ng RNA using a High Capacity cDNA Reverse Transcriptation Kit (Life Technologies) according to the manufacturer’s protocol. 1/50th of the obtained cDNA was used for each qPCR reaction. Reactions were performed in triplicate using TaqMan® Fast Universal PCR Master Mix (Life Technologies) and the StepOnePlus™ Real-Time PCR System (Life Technologies). Gene expression analysis was performed using the Pfaffl method in order to account for qPCR efficiency^40^, whereas the qPCR efficiency was determined by LinRegPCR software^41^. Gene expression levels were normalised to GAPDH.

## Additional Information

**How to cite this article**: Willms, E. *et al*. Cells release subpopulations of exosomes with distinct molecular and biological properties. *Sci. Rep.* **6**, 22519; doi: 10.1038/srep22519 (2016).

## Supplementary Material

Supplementary Information

Supplementary Table S1

Supplementary Table S2

Supplementary Table S3
