# Supplementary material for: Cells release subpopulations of exosomes with distinct molecular and biological properties
Source: Sci Rep. 2016 Mar 2;6:22519. doi: 10.1038/srep22519 (PMC4773763; doi:10.1038/srep22519)
Supplement: Supplementary Information [file srep22519-s1.pdf]

Cells release subpopulations of exosomes with distinct molecular and biological properties

Eduard Willms[1], Henrik J. Johansson[2], Imre Mäger[1,3], Yi Lee[1], K. Emelie M. Blomberg[4], Mariam Sadik[5], Amr Alaarg[1], C.I. Edvard Smith[4], Janne Lehtiö[2], Samir EL Andaloussi[1,4], Matthew J.A. Wood[1,a,]* and Pieter Vader[1,5,a]

[a] Co-senior author

[1] Department of Physiology, Anatomy and Genetics, University of Oxford, Oxford, OX1 3QX, United Kingdom

[2] Cancer Proteomics Mass Spectrometry, Science for Life Laboratory, Department of Oncology-Pathology, Karolinska Institutet, Stockholm, SE-171 21, Sweden

[3] Institute of Technology, University of Tartu, Tartu, 50411, Estonia

[4] Department of Laboratory Medicine, Karolinska Institutet, Stockholm, SE-141 57, Sweden

[5] Department of Clinical Chemistry and Haematology, University Medical Center Utrecht, Utrecht, 3584 CX, The Netherlands

*Corresponding author:

Matthew J.A. Wood

Department of Physiology, Anatomy and Genetics

University of Oxford

Matthew.wood@dpag.ox.ac.uk

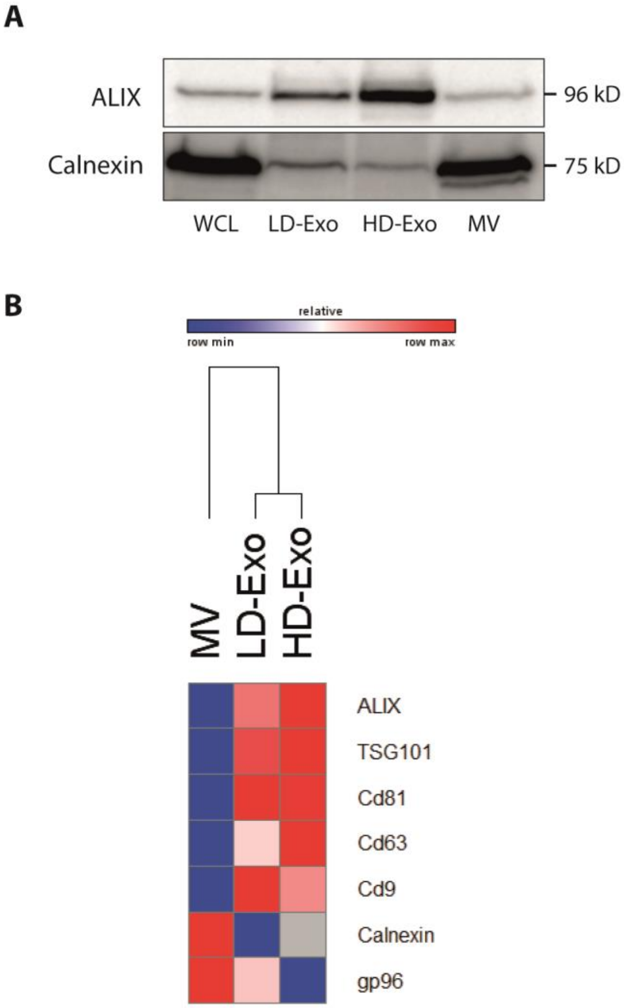

Supplementary Figure S1: Subpopulations of exosomes are enriched in exosome marker proteins, but not endoplasmic reticulum markers, compared to cell lysate and microvesicles. (A) Exosomes were isolated as in Fig 1A. P110 was loaded at the bottom of a sucrose density gradient and subjected to ultracentrifugation for 16 h. Fractions 3-5 (LD fractions) and 8-9 (HD fractions) were analyzed for the presence of Alix and Calnexin by Western blotting. Equal amounts of proteins were analyzed. WCL: Whole cell lysate. (B) Heatmap of representative proteomics data.

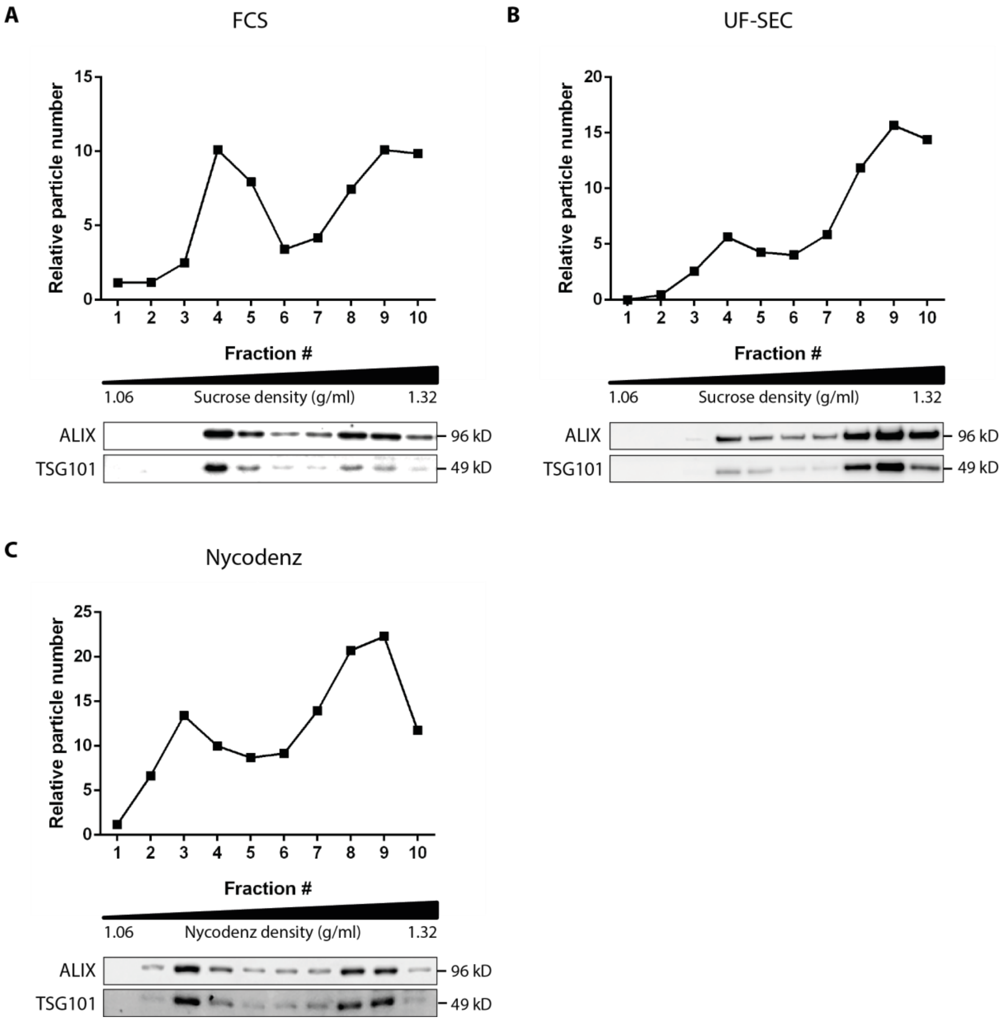

Supplementary Figure S2: Subpopulations of exosomes are released in serum-containing medium and are not a result of the isolation method. (A) Exosomes were collected in medium supplemented with exosome-depleted FCS instead of Opti-MEM and isolated as in Fig 1A. P110 was loaded at the bottom of a sucrose density gradient and subjected to ultracentrifugation for 16 h. (B) Exosomes were isolated using ultrafiltration followed by size-exclusion chromatography instead of ultracentrifugation, loaded at the bottom of a density gradient and subjected to ultracentrifugation for 16 h. (C) Exosomes were isolated as in Fig 1A. P110 was loaded at the bottom of a Nycodenz density gradient instead of a sucrose density gradient, and ultracentrifugated for 16 h. For analysis, the resulting fractions (1-10) with increasing density were analyzed for particle number by NTA (upper panels) and the presence of exosome marker proteins ALIX and TSG101 by Western blotting (lower panels). For Western blots, an equal volume of each sample was analyzed.

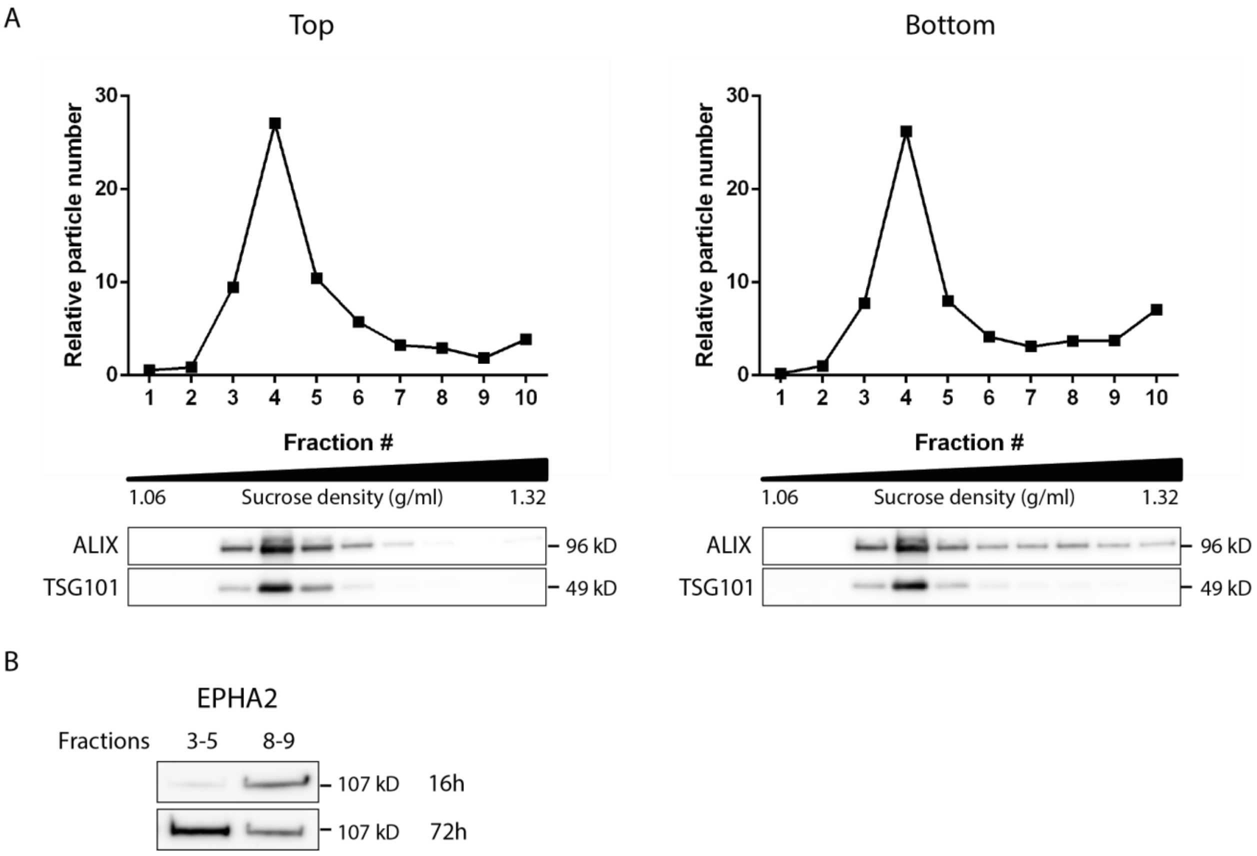

Supplementary Figure S3: Exosome subpopulations equilibrate at the same density. (A) Exosomes were isolated as in Fig 1A. P110 was loaded on top (left panel) or at the bottom (right panel) of a sucrose density gradient and subjected to ultracentrifugation for 72 h. The resulting fractions (1-10) with increasing density were analyzed for particle number by NTA (upper panels) and the presence of exosome marker proteins ALIX and TSG101 by Western blotting (lower panels). (B) P110 was loaded at the bottom of a sucrose density gradient and ultracentrifugated for 16 h or 72 h. Fractions 3-5 (LD fractions) and 8-9 (HD fractions) were analyzed for the presence of EPHA2 by Western blotting. For Western blots, an equal volume of each sample was analyzed.

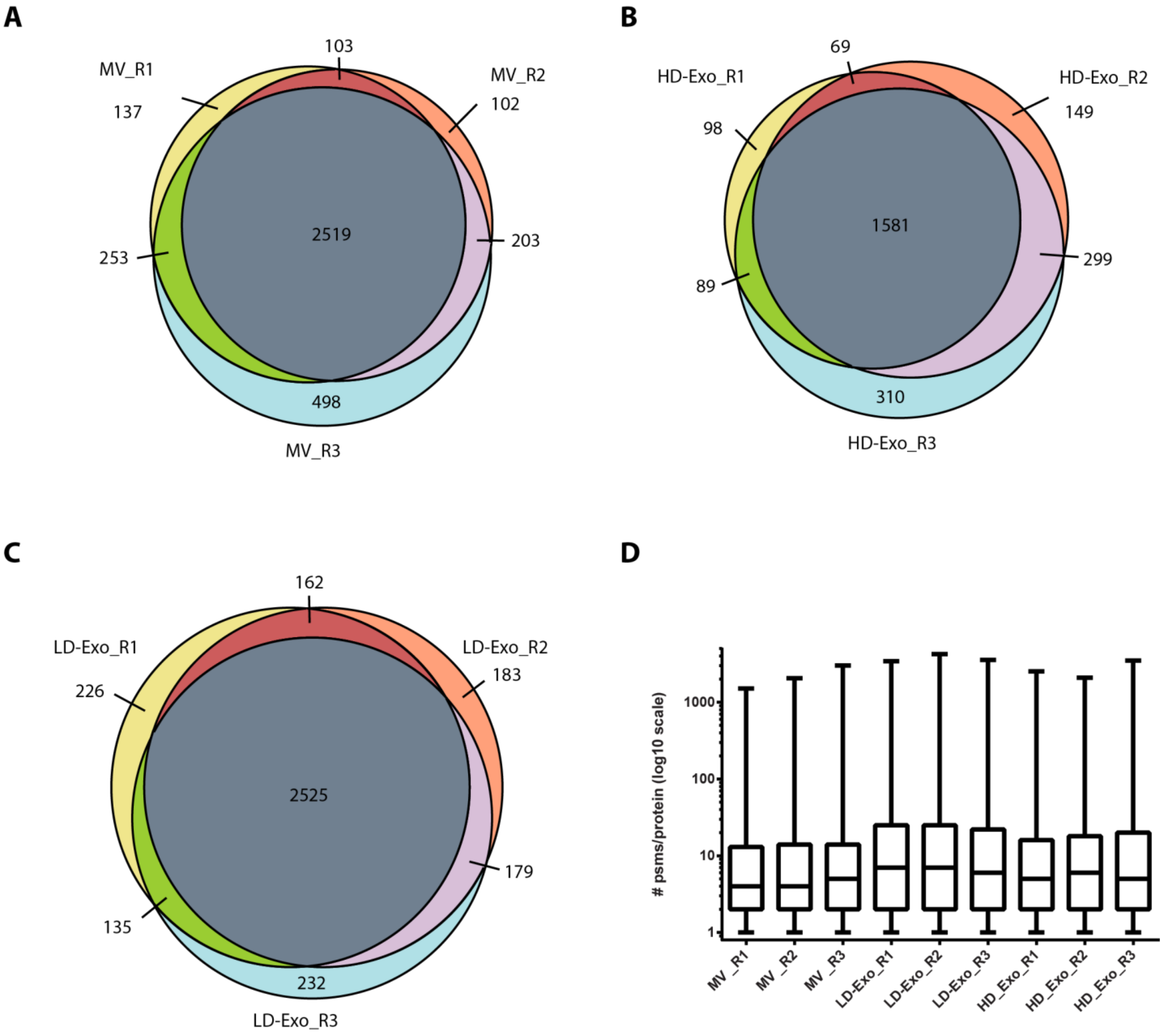

Supplementary Figure S4: Proteomic analysis shows good overlap in number of protein identifications between replicates. Proteomics reproducibility as shown by the protein identification overlap between replicates for (A) MV, (B) HD-Exo, and (C) LD-Exo. (D) Number of psms/protein for identification.

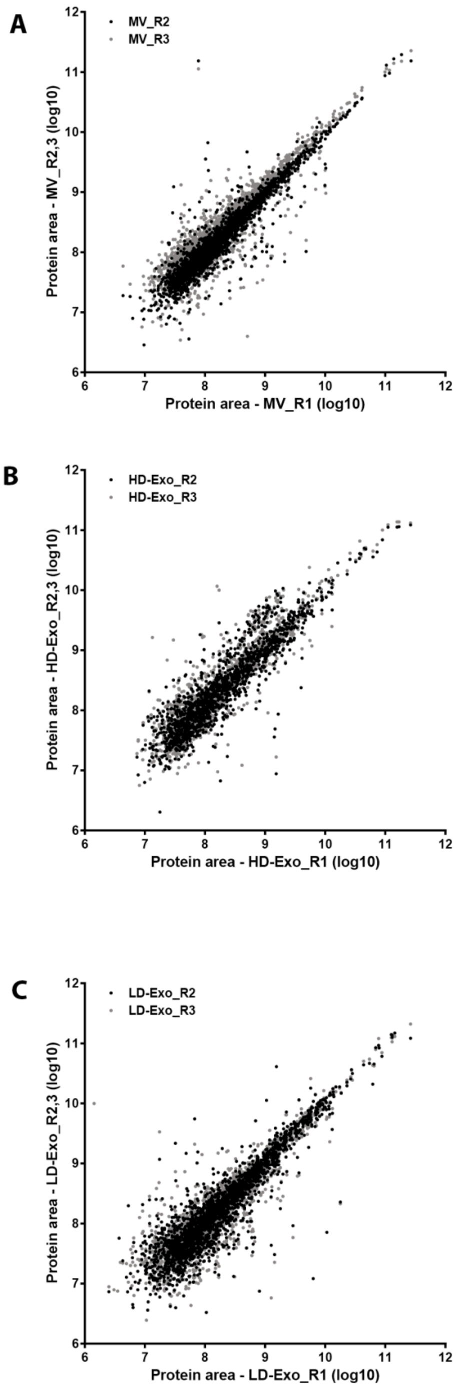

Supplementary Figure S5: Proteomic analysis shows good quantitative reproducibility between replicates. Proteomics reproducibility as shown by the correlation of protein area between replicates for (A) MV, (B) HD-Exo, (C) LD-Exo.

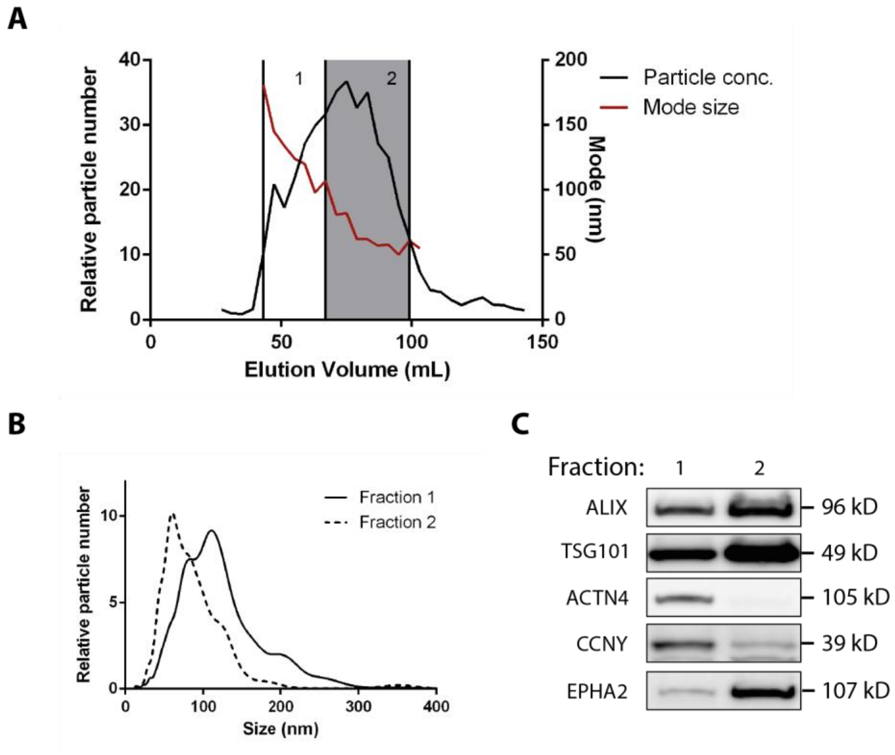

Supplementary Figure S6: ACTN4 and CCNY are enriched in large exosomes, EPHA2 is enriched in small exosomes. Exosomes were isolated as in Fig 1A. P110 was fractionated by SEC using Sephacryl S-1000. The resulting fractions 1and 2 (A) were analyzed for particle size by NTA (B) and the presence of ALIX, TSG101, ACTN4, CCNY and EPHA2 by Western blotting (C). For Western blots, equal amounts of protein were analyzed.

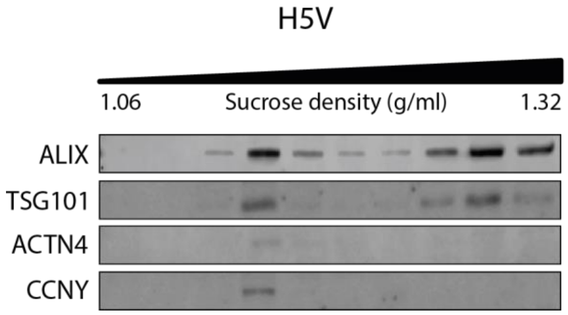

Supplementary Figure S7: ACTN4 and CCNY are enriched in LD-Exo derived from H5V cells. Exosomes were isolated as in Fig 1A. P110 was loaded at the bottom of a sucrose density gradient and subjected to ultracentrifugation for 16 h. The resulting fractions (1-10) with increasing density were analyzed for the presence of ALIX, TSG101, ACTN4 and CCNY by Western blotting. For Western blots, an equal volume of each sample was analyzed.

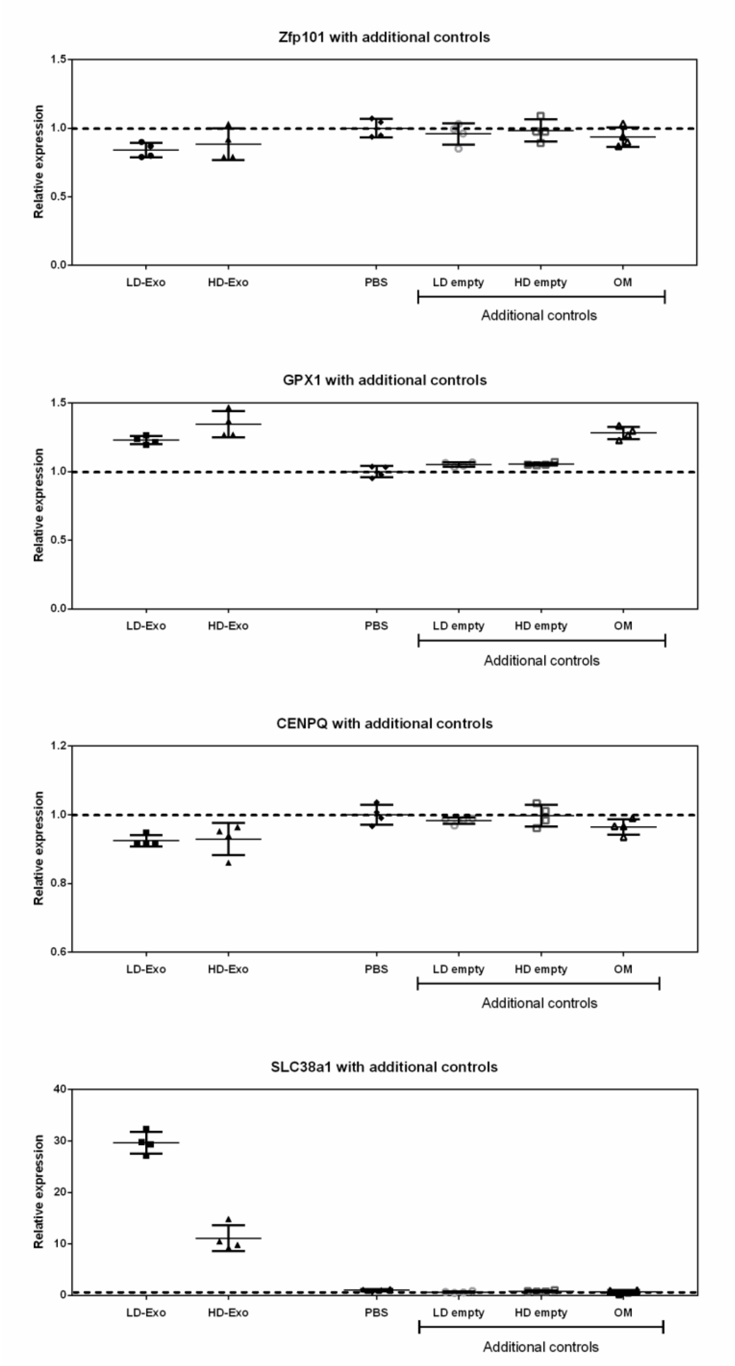

Supplementary Figure S8: Empty LD and HD sucrose fractions have no effect on gene expression in recipient cells. OptiMEM was bottom floated into sucrose, and H5V endothelial cells were subsequently incubated for 24 hours with “empty” sucrose fractions 3-5 and 8-9. As an additional control, OptiMEM was added to the recipient cells directly (OM). Transcript levels were measured relative to GAPDH and plotted relative to levels in PBS-treated cells. Dot plots represent mean ± SD. (n=4).
